# The Comprehensive Repair–Inflammation Index (CRII) Predicts Tooth Extraction After Chemoradiotherapy: A Continuous and Nonlinear Modeling Analysis

**DOI:** 10.3390/jcm15103777

**Published:** 2026-05-14

**Authors:** Erkan Topkan, Efsun Somay, Sibel Bascil, Duriye Ozturk, Ugur Selek

**Affiliations:** 1 Department of Radiation Oncology, Faculty of Medicine, Baskent University, Adana 01120, Turkey; docdretopkan@gmail.com; 2 Department of Oral and Maxillofacial Surgery, Faculty of Dentistry, Baskent University, Ankara 06594, Turkey; 3 Department of Periodontology, Faculty of Dentistry, Baskent University, Ankara 06594, Turkey; bascil5@yahoo.com; 4 Department of Radiation Oncology, School of Medicine, Afyonkarahisar Health Sciences University, Afyonkarahisar 03340, Turkey; duriyeozturk07@gmail.com; 5 Department of Radiation Oncology, School of Medicine, Koc University, Istanbul 34652, Turkey; ugurselek@yahoo.com

**Keywords:** nasopharyngeal neoplasms, chemoradiotherapy, tooth extraction, biomarkers, inflammation, logistic regression

## Abstract

**Background**: Tooth extraction (TE) after chemoradiotherapy is common in locally advanced nasopharyngeal carcinoma (LA-NPC), yet its determinants remain unclear. We evaluated the association between the Comprehensive Repair–Inflammation Index (CRII), reflecting systemic inflammation and host repair capacity, and TE risk after concurrent chemoradiotherapy (CCRT). **Methods**: We conducted a retrospective analysis of 354 patients with LA-NPC treated with definitive CCRT. The primary endpoint was post-treatment TE (none vs. ≥1). CRII was calculated from pre-treatment laboratory parameters and analyzed continuously, with a breakpoint identified via segmented regression. Logistic regression and restricted cubic splines were used. Multivariable models adjusted for clinical variables and mandibular dosimetric parameters (mean dose, V50, V60). **Results**: TE occurred in 70.1% of patients. CRII was significantly higher in those with TE (147.5 vs. 122.0; *p* < 0.001). CRII was strongly associated with TE (per 10-unit increase: OR 1.49, 95% CI 1.34–1.66; *p* < 0.001). A nonlinear relationship was observed, with a breakpoint at 145.7, above which TE rates increased markedly (90.5% vs. 58.8%; *p* < 0.001). CRII remained independently predictive after adjustment (adjusted OR 1.46; ≥145.7: OR 5.1; both *p* < 0.001). Mandibular dose parameters were not significantly associated with tooth extraction in this analysis. **Conclusions**: CRII independently predicts post-CCRT TE with a nonlinear risk pattern, highlighting the potential contribution of systemic host-related factors alongside conventional dosimetric parameters.

## 1. Introduction

Concurrent chemoradiotherapy (CCRT), with or without induction or adjuvant chemotherapy, is a cornerstone in the management of locally advanced nasopharyngeal carcinoma (LA-NPC) [1,2,3,4]. Nevertheless, despite advances in radiotherapy techniques such as intensity-modulated radiotherapy (IMRT) and volumetric modulated arc therapy (VMAT), therapeutic doses of ionizing radiation continue to adversely affect adjacent normal tissues, particularly within the oral cavity, leading to a range of acute and late toxicities that can substantially impair oral health and quality of life [5,6,7,8]. Dental complications are among the most frequent of these effects and often require tooth extraction (TE) during follow-up, with potential consequences including nutritional deficits, weight loss, and impaired functional recovery [8]. Importantly, post-CCRT TE is not merely a routine procedure but represents a clinically consequential event, given its association with impaired wound healing and its recognized role in the development of osteoradionecrosis of the jaw (ORNJ) [9,10,11].

Existing studies of post-CCRT dental outcomes have predominantly adopted a dose-centric framework, in which radiation exposure to the mandible and surrounding structures is considered a principal determinant of tissue injury and subsequent complications [5,6,7]. This paradigm is supported by substantial biological and clinical evidence demonstrating that radiation-induced damage to vascular, epithelial, salivary, and osseous tissues compromises tissue integrity and healing capacity, thereby predisposing to necrosis as well as dental and periodontal deterioration [12,13]. However, clinical outcomes remain variable, and patients with comparable dosimetric profiles frequently exhibit differing risks of TE, suggesting that these outcomes are not fully explained by radiation dose alone [14,15,16].

Beyond this dose-centric perspective, host-related factors may also contribute to inter-individual variability in CCRT-related toxicity. Within this framework, studies evaluating TE after CCRT have yielded inconsistent findings, often relying on dichotomized variables and threshold-based approaches that may obscure underlying continuous risk relationships [14,15,16]. Such approaches typically do not account for the functional form of associations or potential nonlinear effects, and the resulting thresholds are often derived from cohort-specific or data-driven strategies, which may limit their generalizability. In parallel, studies examining radiation dose-related dental injury have primarily described biological or observational dose gradients [5,6], which, while informative, do not fully capture the clinical heterogeneity of dental outcomes, including variability in progression to TE, nor directly support patient-level prediction. Consequently, the extent to which existing approaches capture the complexity of these relationships and support individualized risk estimation remains uncertain.

Building on these considerations, composite indices incorporating host-related factors have been proposed as an approach to characterize inter-individual susceptibility to post-CCRT TE. Previous studies evaluating such composite markers have demonstrated associations with radiation-induced dental complications in patients with LA-NPC [14,15,16]. However, these studies have predominantly relied on categorized or cutoff-based analyses. They have not evaluated associations within a continuous modeling framework or formally assessed potential nonlinear effects. According to established statistical guidance, such approaches may obscure underlying risk gradients and limit the ability to capture the complexity of biological responses [17,18,19]. Whether a composite repair–inflammation index can improve the characterization of post-CCRT TE risk within a continuous, nonlinear modeling framework, therefore, remains unknown.

Accordingly, this study evaluated the association between the Comprehensive Repair–Inflammation Index (CRII)—defined as (platelet × neutrophil × monocyte)/(lymphocyte × hemoglobin × albumin)—and post-CCRT TE in a retrospective cohort of patients with LA-NPC treated with definitive CCRT. Specifically, we aimed to characterize the continuous and potentially nonlinear relationship between CRII and TE risk and to examine this association in relation to conventional mandibular radiation dose parameters.

## 2. Patients and Methods

### 2.1. Study Population

Institutional records from the Başkent University Adana Research and Treatment Center were retrospectively reviewed to identify patients with LA-NPC who underwent definitive CCRT and received oral and dental evaluations before treatment between January 2010 and December 2021. Eligible patients were aged ≥18 years and had histopathologically confirmed squamous cell carcinoma. All patients were classified as having locally advanced disease according to the American Joint Committee on Cancer (AJCC) 8th edition staging system. Additional inclusion criteria included an Eastern Cooperative Oncology Group (ECOG) performance status of 0–1, no prior malignancy, no previous radiotherapy or systemic chemotherapy to the head and neck region, receipt of platinum-based CCRT, and availability of pre-treatment complete blood count and biochemistry data. To ensure accurate assessment of dental outcomes, patients were also required to have accessible pre-treatment and follow-up dental records, including panoramic and/or periapical radiographic examinations and documented clinical oral evaluations.

**Figure 1:**
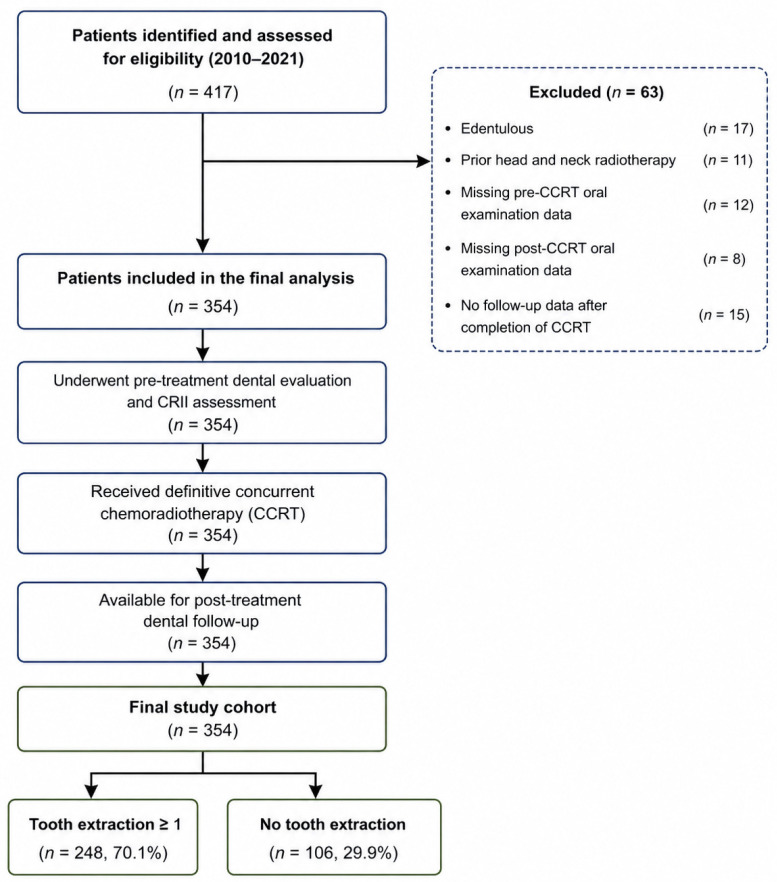
Flow diagram of patient selection. Note: A total of 417 patients were assessed for eligibility. After application of predefined exclusion criteria, 354 patients were included in the final analysis. The cohort was stratified according to the occurrence of post-treatment tooth extraction (*n* = 248) versus no extraction (*n* = 106). **Abbreviations:** CCRT, concurrent chemoradiotherapy; CRII, Comprehensive Repair–Inflammation Index.

Patients were excluded if they had complete edentulism, tumor or lymph node invasion involving the mandible, prior history of jaw surgery, use of systemic steroids or immunomodulatory agents within 30 days before initiation of CCRT, receipt of blood transfusion within 30 days prior to treatment, or presence of active systemic inflammatory or immunological disorders. Patients with traumatic or non-treatment-related tooth loss during follow-up were also excluded. From an initial cohort of 417 patients identified during the study period, 63 were excluded based on these criteria (Figure 1), resulting in a final study population of 354 patients.

### 2.2. Baseline Clinical Oral Examination

Pre-treatment oral and dental status was systematically assessed using standardized clinical and radiographic evaluations. Data extracted from medical records included dental and periodontal conditions such as facial swelling, toothache, gingival edema, abscess formation, dental caries, and pain on percussion. All evaluations were performed by an experienced oral and maxillofacial surgeon in collaboration with a dentomaxillofacial radiologist. Detailed dental parameters were recorded to characterize baseline oral health status, including the total number of teeth, the number of decayed teeth (crown and/or root caries), the presence of residual roots, the history of periodontal and endodontic treatments, and the number of filled or previously extracted teeth. These variables were considered in the context of potential susceptibility to post-CCRT tooth extraction. Radiographic evaluation was performed using panoramic and periapical imaging according to institutional protocols. Panoramic radiographs were obtained using a standardized digital system (Veraviewepocs 2D, J Morita, Kyoto, Japan), and periapical radiographs were acquired using the parallel technique with a digital intraoral system. Imaging acquisition was conducted under consistent conditions across all patients in accordance with manufacturer recommendations to ensure comparability.

### 2.3. Assessment of the Comprehensive Repair–Inflammation Index (CRII)

The CRII was calculated as follows:

$$CRII=\frac{platelet\;\times \;neutrophil\;\times \;monocyte}{lymphocyte\;\times \;hemoglobin\;\times \;albumin}$$

For interpretability, CRII values were scaled by a factor of 10^−3^ (i.e., divided by 1000) prior to analysis; this scaled variable was used consistently across all statistical models, including logistic regression, restricted cubic spline modeling, and segmented regression, and is presented accordingly throughout the manuscript.

All component variables were obtained from routine laboratory measurements performed on the first day of CCRT prior to treatment initiation. Hematological parameters (platelet, neutrophil, monocyte, and lymphocyte counts) were recorded in ×10^9^/L, hemoglobin in g/dL, and albumin in g/L, in accordance with standard clinical practice. This approach ensured a standardized, treatment-naïve assessment of systemic inflammatory burden and host-related physiological status across all patients. Laboratory measurements were performed as part of routine clinical care using institutional protocols, and CRII values were computed directly from these measurements with only scaling applied for presentation. No further normalization or transformation was applied.

### 2.4. Chemoradiotherapy Protocol

Radiotherapy was delivered using a simultaneous integrated boost IMRT (SIB-IMRT) technique. Target volumes were delineated based on pre-treatment co-registered computed tomography (CT), 18F-fluorodeoxyglucose positron emission tomography–CT (FDG PET-CT), and/or magnetic resonance imaging (MRI) of the primary tumor and neck regions.

Prescribed doses were 70 Gy to high-risk, 59.4 Gy to intermediate-risk, and 54 Gy to low-risk planning target volumes (PTVs), delivered in 33 fractions over approximately 6.5 weeks (once daily, 5 days per week), in accordance with institutional protocols [20].

Concurrent chemotherapy consisted of weekly cisplatin administered at a dose of 40 mg/m^2^ for up to seven cycles during RT. Following completion of CCRT, patients were recommended to receive two cycles of adjuvant chemotherapy with cisplatin and 5-fluorouracil, based on clinical suitability.

Supportive care measures, including antiemetic prophylaxis, hydration, nutritional support, and other interventions as clinically indicated, were provided throughout treatment.

### 2.5. Follow-Up Dental Examination

Follow-up oral and dental assessments were performed using the same standardized clinical and radiographic procedures described for the baseline evaluation. Examinations were conducted at 1, 3, 6, 9, and 12 months after completion of CCRT, and subsequently at 6-month intervals or as clinically indicated.

Clinical and radiological findings were systematically recorded at each follow-up visit. Dental management decisions were made in accordance with the principles applied at baseline, ensuring consistency in assessment and treatment approaches throughout the follow-up period.

### 2.6. Indications for Post-CCRT Tooth Extraction

Post-CCRT TE was performed based on clinical and radiographic findings indicating poor dental prognosis. Indications included non-restorable caries, residual roots, persistent or symptomatic apical or periodontal infection, advanced periodontal breakdown, severe mobility, pain on percussion, abscess formation, and teeth considered non-maintainable despite conservative management. Extraction decisions were made by the treating dental team based on predefined clinical and radiographic criteria, following comprehensive evaluation, with consideration of impaired healing capacity associated with irradiated tissues. These criteria were applied consistently across patients within the same institutional framework to ensure uniformity in decision-making.

### 2.7. Endpoints and Statistical Analysis

The primary endpoint was post-CCRT TE, defined as a binary outcome (no TE vs. ≥1 TE). Continuous variables are presented as mean ± standard deviation, and categorical variables as counts and percentages. Between-group comparisons were performed using the independent-samples *t*-test or the χ^2^ test, as appropriate. The association between the CRII and TE was evaluated using logistic regression, with results reported as odds ratios (ORs) and 95% confidence intervals (CIs). A logistic approach was chosen given the study design and the absence of precise event-time data for all patients, which precluded reliable time-to-event analyses. CRII was modeled as a continuous variable in the primary analysis (per 1-unit increase), with additional estimates per 10-unit increase provided to enhance clinical interpretability. Potential nonlinearity was assessed using restricted cubic splines (three knots), with model comparisons performed using likelihood ratio tests, and segmented logistic regression was applied to identify an exploratory breakpoint in the CRII–TE relationship for subsequent descriptive stratified analyses.

Multivariable models were constructed to evaluate the independent association of CRII with TE, adjusting for age, sex, smoking status, alcohol use, and mandibular radiation dose parameters, including mean dose and high-dose volume metrics (V50 and V60, defined as ≥1 cc vs. <1 cc). Mandibular V50 and V60 were evaluated based on prior evidence supporting the relevance of dose–volume exposure in the 50–60 Gy range [21]. Given the limited distribution of high-dose mandibular volumes in this cohort, these variables were dichotomized at ≥1 cc versus <1 cc to represent the presence versus absence of appreciable high-dose exposure; this threshold was used pragmatically to ensure model stability and was not intended to define a biological or clinical cutoff.

Covariates were selected a priori based on clinical relevance and constrained by the number of observed events to minimize the risk of overfitting. No data-driven variable selection procedures (e.g., stepwise selection) were applied, and model complexity was deliberately limited to ensure parsimony. Potential effect modification was evaluated using interaction terms, and collinearity among predictors was assessed. A formal a priori sample size calculation was not performed due to the retrospective design; model size was instead guided by the number of observed events. Model calibration was evaluated using the Hosmer–Lemeshow goodness-of-fit test. No missing data were present for variables included in the final models; therefore, complete-case analysis was applied. All analyses were performed using IBM SPSS Statistics (version 26.0) and R software (version 4.5.1). All tests were two-sided, and *p*-values < 0.05 were considered statistically significant.

This study was conducted and reported in accordance with the Strengthening the Reporting of Observational Studies in Epidemiology (STROBE) guidelines for observational studies, and a completed STROBE checklist is provided as Supplementary Table S1 [21].

## 3. Results

### 3.1. Patient Characteristics and Group Comparisons

A total of 354 patients were included in the final analysis (Figure 1), of whom 248 (70.1%) underwent at least one post-chemoradiotherapy tooth extraction (TE), while 106 (29.9%) did not. Baseline characteristics are summarized in Table 1. The median follow-up duration was 64.3 months (range, 7.4–146.7 months). The median time to first tooth extraction was 16.3 months (range, 8.1–109.4 months) after completion of CCRT. The majority of extraction events occurred within the first 24 months (64.9%), with a declining frequency thereafter. There were no significant differences between groups with respect to baseline demographic, clinical, and treatment-related variables, including age, sex, ECOG performance status, smoking status, alcohol use, diabetes status, tumor stage (T and N), histology, and mandibular mean radiation dose (Table 1). In contrast, CRII values were significantly higher in patients who underwent TE compared with those who did not (147.5 ± 22.0 vs. 122.0 ± 29.4, *p* < 0.001), consistent with the strong association observed in regression analyses (Table 2).

### 3.2. Association Between CRII and Tooth Extraction

When modeled as a continuous variable, CRII was strongly associated with TE. Each 1-unit increase in CRII was associated with increased odds of TE (OR 1.041, 95% CI 1.030–1.053; *p* < 0.001), corresponding to an OR of 1.49 (95% CI 1.34–1.66; *p* < 0.001) per 10-unit increase (Table 2).

### 3.3. Nonlinear Modeling and Exploratory Threshold Analysis

**Figure 2:**
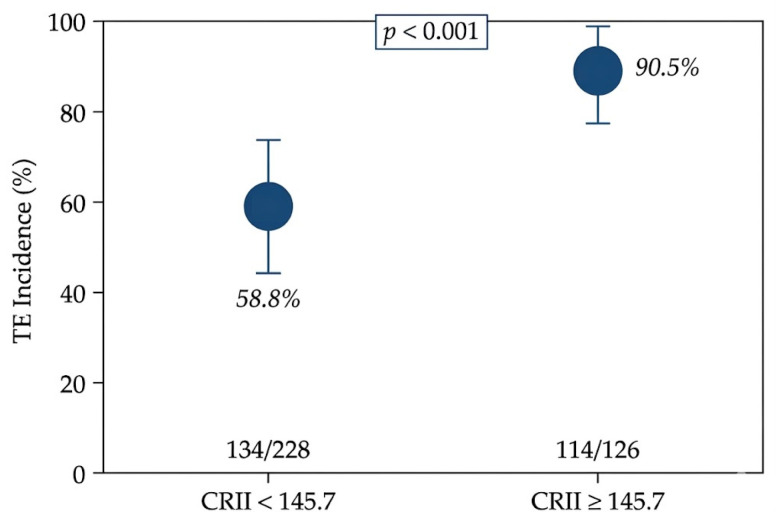
Incidence of post-CCRT tooth extraction according to CRII cutoff. **Note:** Bars represent incidence proportions, with 95% Wilson confidence intervals. Extraction rates were higher in patients with CRII ≥145.7 than in those below the threshold (90.5% vs. 58.8%; *p* < 0.001). Numbers within bars indicate event counts.

Assessment of the functional form using restricted cubic splines indicated deviation from linearity compared with a linear model (χ^2^ = 8.18, df = 3; *p* = 0.042). Segmented logistic regression identified an exploratory breakpoint at a CRII value of 145.7, with modest improvement in model fit (χ^2^ = 4.66, df = 1; *p* = 0.031) (Table 2). Stratification based on this cutoff showed a marked difference in TE incidence, with rates of 58.8% (134/228) for CRII <145.7 and 90.5% (114/126) for CRII ≥ 145.7 (*p* < 0.001), corresponding to an unadjusted OR of 6.6 (95% CI 3.5–12.4) (Table 2; Figure 2).

### 3.4. Multivariable and Interaction Analyses

In multivariable models adjusting for age, sex, smoking status, alcohol use, and mandibular radiation dose parameters, CRII remained independently associated with TE. The effect estimate was minimally attenuated (adjusted OR per 1-unit increase 1.038, 95% CI 1.026–1.050; *p* < 0.001), with a corresponding adjusted OR of 5.1 (95% CI 2.5–10.4; *p* < 0.001) for CRII ≥ 145.7. None of the adjustment covariates (age, sex, smoking status, and alcohol use) were significantly associated with TE in either univariable or multivariable analyses (Table 2). The model demonstrated adequate calibration based on the Hosmer–Lemeshow test (*p* > 0.05), and no evidence of multicollinearity was observed among included covariates (all variance inflation factors < 1.5).

Mandibular dose parameters were not significantly associated with TE, including mandibular mean dose (per 1 Gy increase: OR 0.997, 95% CI 0.975–1.018; *p* = 0.756) and high-dose volume metrics (V50 ≥ 1 cc and V60 ≥ 1 cc; all *p* > 0.20) (Table 2). No significant interaction was observed between CRII and mandibular dose parameters (all interaction *p*-values > 0.20), indicating no evidence of effect modification (Supplementary Table S2).

**Figure 3:**
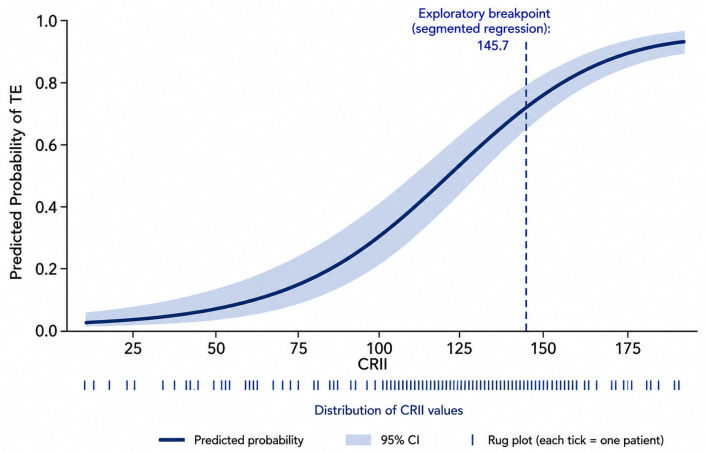
Predicted probability of post-CCRT tooth extraction according to CRII. Note: The curve shows the estimated probability from logistic regression with CRII modeled as a continuous variable. The shaded area represents the 95% confidence interval, and tick marks indicate the distribution of CRII values. The dashed vertical line denotes the exploratory breakpoint (segmented regression) at CRII 145.7. The relationship demonstrates a progressive increase in extraction risk across the CRII spectrum, consistent with a nonlinear association.

The continuous relationship between CRII and TE probability is illustrated in Figure 3, demonstrating a progressive increase in predicted risk across the CRII spectrum, with higher probabilities observed beyond the exploratory breakpoint.

## 4. Discussion

In this study, CRII demonstrated a robust association with post-CCRT TE, with consistent findings across multiple analytical approaches. The relationship between CRII and TE was not strictly linear; evidence of a nonlinear pattern and an exploratory threshold was observed, beyond which TE risk increased substantially. This association persisted after adjustment for clinical and dosimetric variables. Within this framework, tooth extraction should be interpreted as a clinically meaningful composite endpoint reflecting both biological tissue vulnerability and real-world decision-making processes in the management of irradiated dentition. Mandibular radiation dose parameters were not significantly associated with TE, and no interaction was observed between CRII and dose metrics. These findings suggest that the association between CRII and TE was not materially modified by radiation exposure within the constraints of the present analysis. Notably, conventional clinical variables, including tumor stage and performance status, were not associated with TE. This suggests that systemic host-related factors captured by CRII may contribute to inter-individual variability. CRII should be interpreted as an integrative composite index that builds upon established inflammation- and nutrition-based biomarkers rather than as a distinct construct. Collectively, these findings support a role for systemic host-related factors, as reflected by CRII, alongside clinical and dosimetric parameters in shaping TE risk.

The association between CRII and tooth extraction was consistent across multiple analytical approaches, including continuous modeling, nonlinear assessment, and exploratory stratification, with minimal attenuation after multivariable adjustment. This internal consistency supports the robustness of the association, suggesting that the observed effect is not driven by a specific modeling strategy. Notably, the effect remained stable after accounting for clinical characteristics and mandibular dose parameters, indicating that CRII captures information not accounted for by conventional clinical and dosimetric variables. The observed gradient in risk across the CRII spectrum, together with the marked separation in tooth extraction incidence based on the exploratory cutoff (58.8% vs. 90.5%, *p* < 0.001), further reinforces the coherence of the association across analytical frameworks. Although the present study was not designed to disentangle the individual contributions of CRII components, the index integrates multiple dimensions of systemic physiology. These include inflammatory burden, immune competence, nutritional reserve, and oxygen-carrying capacity, all of which are biologically relevant to tissue repair and response to radiation injury [12,22,23,24,25,26]. Within this context, CRII may be conceptualized as a composite indicator of host-related physiological reserve relevant to tissue repair rather than a surrogate for any single biological pathway.

Previous studies evaluating TE after CCRT have reported heterogeneous findings regarding the contribution of radiation dose, influenced by differences in dosimetric granularity and the incorporation of local clinical factors. Experimental and clinical data suggest that radiation-induced dental injury follows dose-dependent patterns, with minimal effects at lower doses and progressive structural damage at higher exposure levels [5,6]. These biological observations are also consistent with mechanisms implicated in ORNJ, although most available evidence is derived from tooth- or tissue-specific dose assessments rather than organ-level mandibular metrics. Studies specifically addressing TE have yielded inconsistent results: in one analysis, mandibular dose parameters remained significant alongside a systemic biomarker, suggesting a combined contribution of local and systemic factors [14], whereas in another, dose variables were not retained and a systemic inflammation index emerged as the primary determinant of TE risk [15]. Notably, composite indices such as the GLUCAR index, systemic immune–inflammation index (SII), and CARWL index have demonstrated predictive value for radiation-related dental outcomes [14,15,16]. More broadly, systemic inflammation-based indices have been consistently associated with prognosis and treatment outcomes across multiple cancer types, supporting the relevance of host-related systemic factors in oncologic risk stratification [27,28,29].

However, these indices primarily capture inflammatory or metabolic dimensions and do not integrate parameters reflecting host-related physiological reserve relevant to tissue repair, nutritional status, and oxygen-carrying capacity within a single framework. In addition, prior studies have predominantly relied on cutoff-based approaches with dichotomized variables, which may limit characterization of continuous and potentially nonlinear risk relationships. In the present study, using continuous modeling with formal assessment of nonlinearity, mandibular dose parameters—including mean dose and commonly used high-dose volume metrics (V50 and V60)—were not associated with TE, and no interaction was observed between CRII and these measures. These parameters reflect mandibular exposure at the organ level and may not capture focal dose heterogeneity relevant to individual teeth or extraction sites. Within these constraints, CRII demonstrated a consistent association across all analytical models, including after adjustment for dosimetric variables, suggesting that systemic host-related factors—integrating inflammatory burden, immune competence, nutritional status, and oxygenation—may contribute to inter-individual variability in TE risk beyond that captured by conventional dose metrics or single-domain composite indices. This pattern is illustrated in Figure 3, supporting the observed separation in TE risk across the CRII threshold. Importantly, this interpretation does not diminish the biological relevance of radiation exposure but indicates that, within the context of the present analysis, host-related factors provide additional explanatory insight.

Beyond demonstrating an independent association, the present analysis provides important insight into the functional form of the relationship between CRII and TE. Continuous modeling demonstrated a monotonic increase in TE risk across the CRII spectrum, with formal testing indicating deviation from strict linearity (spline vs. linear: χ^2^ = 8.18, df = 3, *p* = 0.042; segmented vs. linear: χ^2^ = 4.66, df = 1, *p* = 0.031), supporting a nonlinear risk pattern rather than a simple proportional effect. Within this context, the exploratory cutoff identified through data-driven methods provides a pragmatic illustration of risk stratification, with a marked separation in TE incidence above versus below this threshold (58.8% vs. 90.5%, *p* < 0.001). However, consistent with established statistical guidance, this cutoff should be interpreted as descriptive rather than definitive, as dichotomization entails information loss and may generate cohort-specific thresholds [17,18]. Prior studies evaluating TE outcomes, including those incorporating GLUCAR, systemic immune–inflammation indices, and the CARWL index, have largely relied on cutoff-based approaches without formal assessment of continuous or nonlinear relationships; accordingly, their reported thresholds are best interpreted as exploratory rather than generalizable risk boundaries [14,15,16]. A related but distinct limitation applies to studies describing dose-dependent dental injury, such as those by Walker et al. and Klarić Sever et al., where reported dose ranges reflect biological gradients derived from experimental or observational data rather than patient-level predictive modeling for TE, limiting their direct applicability to individualized risk estimation [5,6]. More recently, studies incorporating tooth-level dosimetry have improved characterization of spatial heterogeneity in radiation exposure [30,31]; however, these approaches remain largely descriptive and have not been integrated with patient-level predictive modeling or continuous risk estimation frameworks. In contrast, the present study integrates continuous modeling, spline-based nonlinearity assessment, and exploratory breakpoint analysis within a unified framework, enabling both precise estimation of risk gradients and cautious identification of clinically interpretable thresholds. Within this framework, CRII demonstrated a consistent and biologically plausible increase in TE risk across its range, with the cutoff serving as an adjunct to, rather than a substitute for, continuous risk modeling.

These findings suggest that CRII may serve as a practical tool for risk stratification in patients undergoing CCRT, enabling identification of patients at increased risk for post-treatment TE. The observed gradient in risk across the CRII spectrum, supported by both continuous modeling and exploratory stratification, indicates that CRII captures meaningful variability not reflected by the evaluated conventional mandibular dosimetric parameters. In this context, CRII may help inform the intensity of dental surveillance, the timing of preventive interventions, and the prioritization of supportive care measures during follow-up, particularly in patients without clearly elevated dosimetric risk. Although mandibular dose metrics were not significantly associated with TE in the present analysis, this finding should be interpreted within the constraints of organ-level dosimetric assessment, which may not capture tooth- or socket-specific dose heterogeneity relevant to individual outcomes. Accordingly, reliance on conventional dosimetric parameters alone may be insufficient for individualized risk assessment, and incorporation of systemic host-related factors may provide complementary clinical insight. However, given the retrospective design and the exploratory nature of the identified cutoff, these findings should be interpreted with caution and not used as a standalone basis for clinical decision-making. Rather, CRII should be considered a complementary risk indicator that may assist in identifying patients who could benefit from closer monitoring and targeted preventive strategies. Prospective validation, together with integration of more granular dosimetric approaches—particularly tooth- or socket-level dose assessment—will be essential to refine its clinical applicability and define its role within multidisciplinary care pathways. Given the established role of post-radiotherapy TE as a trigger for ORNJ, CRII-based risk stratification may also have indirect relevance for identifying patients at increased risk of this complication and informing preventive strategies, although this requires validation in outcome-specific studies.

Several limitations should be explicitly acknowledged. These limitations relate to study design, measurement granularity, and model generalizability. First, the retrospective design introduces the potential for residual confounding and limits causal inference, despite the use of multivariable modeling and interaction analyses. Second, although comprehensive clinical and treatment-related variables were incorporated, residual confounding from unmeasured or more granular oral health–related factors cannot be excluded. Unmeasured factors—particularly those related to baseline dental status, tooth-level restorability, periodontal status, oral hygiene behaviors, and provider-level decision-making—may have influenced the indication for tooth extraction. Although all patients underwent pre-treatment dental evaluation, including extraction of non-restorable teeth, and received standardized dental support, these more granular oral health variables were not systematically captured. Therefore, they could not be included in the multivariable model. As a result, both underlying biological susceptibility and variability in clinical decision-making may have contributed to the observed associations. The relatively high rate of post-CCRT tooth extraction in this cohort may reflect institutional practice patterns, including systematic dental surveillance and a proactive extraction strategy designed to mitigate the risk of infectious complications and ORNJ. In addition, differences in baseline oral health status and oral care practices across populations may have contributed to the observed event rate, potentially limiting direct comparability with other cohorts. Accordingly, this high event rate should be interpreted in the context of these factors and the composite nature of the endpoint, rather than as a direct reflection of underlying biological susceptibility alone. Third, the dosimetric evaluation was based on mandibular-level parameters, which do not capture spatial heterogeneity at the level of individual teeth or extraction sites. In addition, mandibular dose–volume parameters (V50 and V60) were dichotomized at ≥1 cc as a pragmatic representation of appreciable high-dose exposure; although this approach facilitated model stability in the context of limited high-dose volume distribution, it does not represent a validated biological threshold. As such, the absence of a significant dose–response relationship in the present analysis should be interpreted within the constraints of the available dosimetric resolution. Fourth, while the exploratory cutoff for CRII provided clinically intuitive stratification, it remains cohort-specific and requires external validation before broader application. Fifth, CRII was derived from a single pre-treatment measurement, whereas its components may fluctuate during and after chemoradiotherapy, potentially altering its relationship with tooth extraction over time; future studies incorporating serial assessments and time-updated modeling may better capture these dynamics. Finally, although the number of events was sufficient for the primary analyses, it may limit the stability of more complex modeling strategies and subgroup evaluations; therefore, larger cohorts or pooled datasets will be required to support more granular analyses.

Future research should focus on prospective, multicenter validation and on integrative modeling frameworks that combine systemic biomarkers with refined dosimetric and clinical data to improve individualized risk prediction. In particular, approaches that incorporate dynamic assessment of host-related factors together with spatially resolved radiation exposure may enhance the precision and clinical applicability of risk stratification. Such efforts may facilitate more personalized preventive strategies and follow-up planning in patients undergoing CCRT.

## 5. Conclusions

In conclusion, CRII was independently associated with post-CCRT TE, demonstrating a consistent relationship across both continuous and nonlinear modeling approaches. This relationship was not significantly associated with the evaluated conventional mandibular dosimetric parameters in the present analysis. These findings suggest that systemic host-related factors may contribute to TE risk alongside dosimetric parameters. While these results highlight the potential value of integrated, host-informed risk assessment approaches, further external validation is required before clinical application.

## Figures and Tables

**Table 1:** Baseline demographic, clinical, and treatment characteristics of the study cohort according to post-chemoradiotherapy tooth extraction (TE) status.

Variable	Total (*n* = 354)	No TE (*n* = 106)	TE ≥ 1 (*n* = 248)	*p*-Value †
Age, years (mean ± SD)	55.7 ± 13.6	55.7 ± 13.6	54.3 ± 12.9	0.367
Sex, *n* (%) Male Female	284 (80.2) 70 (19.8)	86 (81.1) 20 (18.9)	198 (79.8) 50 (20.2)	0.732
ECOG performance status, *n* (%) 0 1	168 (47.5) 186 (52.5)	52 (49.1) 54 (50.9)	116 (46.8) 132 (53.2)	0.607
Smoking status, *n* (%) Never Ever	35 (9.9) 319 (90.1)	10 (9.4) 96 (90.6)	25 (10.1) 223 (89.9)	0.781
Alcohol use, *n* (%) Never Ever	140 (39.5) 214 (60.5)	41 (38.7) 65 (61.3)	99 (39.9) 149 (60.1)	0.740
Diabetes mellitus, *n* (%) Absent Present	301 (85.0) 53 (15.0)	91 (85.8) 15 (14.2)	210 (84.7) 38 (15.3)	0.864
WHO histology, *n* (%) Type 2 Type 3	37 (10.4) 317 (89.6)	10 (9.4) 96 (90.6)	27 (10.9) 221 (89.1)	0.624
T stage, *n* (%) T1–2 T3–4	60 (16.9) 294 (83.1)	19 (17.9) 87 (82.1)	41 (16.5) 207 (83.5)	0.807
N stage, *n* (%) N0– N2–3	109 (30.8) 245 (69.2)	34 (32.1) 72 (67.9)	75 (30.2) 173 (69.8)	0.679
Mandibular mean dose, Gy (mean ± SD)	35.4 ± 10.4	35.7 ± 10.2	35.3 ± 10.6	0.753
CRII (mean ± SD)	135.8 ± 26.8	122.0 ± 29.4	147.5 ± 22.0	<0.001
CRII category, *n* (%) <145.7 ≥145.7	228 (64.4) 126 (35.6)	94 (88.7) 12 (11.3)	134 (54.0) 114 (46.0)	<0.001

**Note:** † Continuous variables were compared using the independent samples *t*-test, and categorical variables were compared using the χ^2^ test or Fisher’s exact test, as appropriate. **Abbreviations:** CRII, Comprehensive Repair–Inflammation Index; ECOG, Eastern Cooperative Oncology Group; SD, standard deviation; TE, tooth extraction; WHO, World Health Organization.

**Table 2:** Association between the Comprehensive Repair–Inflammation Index (CRII) and post-chemoradiotherapy tooth extraction (TE) in patients with locally advanced nasopharyngeal carcinoma (LA-NPC).

Analysis	Variable/Group	TE (Events/Total)	TE İncidence (%)	Univariate OR (95% CI)	*p*-Value ‡	Multivariate OR (95% CI)	*p*-Value ‡	β (SE)	χ^2^ (df)
Continuous (primary)	CRII (continuous, per 1-unit increase)	—	—	1.041 (1.030–1.053)	<0.001	1.038 (1.026–1.050)	<0.001	0.040 (0.005)	—
	CRII (continuous, per 10-unit increase)	—	—	1.49 (1.34–1.66)	<0.001	1.45 (1.29–1.63)	<0.001	—	—
Exploratory cutoff	CRII <145.7	134/228	58.8	Reference	—	Reference	—	—	—
	CRII ≥145.7	114/126	90.5	6.6 (3.5–12.4)	<0.001	5.1 (2.5–10.4)	<0.001	—	—
Nonlinearity testing	Spline vs. linear	—	—	—	—	—	—	—	8.18 (3)
	Segmented vs. linear	—	—	—	—	—	—	—	4.66 (1)
Dose variables	Mandibular mean dose (continuous, per 1 Gy)	—	—	0.997 (0.975–1.018)	0.756	0.998 (0.976–1.020)	0.782	—	—
	Mandibular mean dose (continuous, per 5 Gy)	—	—	0.99 (0.88–1.10)	0.748	0.99 (0.88–1.11)	0.769	—	—
	Mandibular V50 (≥1 cc)	—	—	1.50 (0.78–2.86)	0.222	1.42 (0.72–2.79)	0.308	—	—
	Mandibular V60 (≥1 cc)	—	—	1.08 (0.37–3.11)	0.890	1.05 (0.36–3.06)	0.932	—	—
Adjustment covariates	Age (years)	—	—	0.992 (0.974–1.009)	0.357	0.996 (0.976–1.016)	0.666	—	—
	Sex (male vs. female)	—	—	0.603 (0.358–1.013)	0.056	0.65 (0.338–1.082)	0.090	—	—
	Smoking status (ever vs. never)	—	—	0.906 (0.558–1.471)	0.688	0.868 (0.503–1.499)	0.613	—	—
	Alcohol use (ever vs. never)	—	—	1.112 (0.700–1.765)	0.653	1.165 (0.685–1.980)	0.573	—	—

**Note:** CRII was analyzed as a continuous variable (per 1-unit and per 10-unit increase) and using an exploratory cutoff derived from segmented logistic regression. Odds ratios (ORs) for both per 1-unit and per 10-unit increases are presented separately for univariate and multivariable models in the corresponding columns. Nonlinearity was assessed using restricted cubic splines (χ^2^ = 8.18, df = 3; *p* = 0.042), and segmented regression identified an exploratory breakpoint at CRII = 145.7 (χ^2^ = 4.66, df = 1; *p* = 0.031). Multivariable models were adjusted for age, sex, smoking status, alcohol use, and mandibular radiation dose parameters. No evidence of multicollinearity was observed among included covariates (all variance inflation factors < 1.5). Model calibration was adequate based on the Hosmer–Lemeshow goodness-of-fit test (*p* > 0.05). ‡ Two-sided *p*-values were derived from logistic regression models. **Abbreviations:** CRII, Comprehensive Repair–Inflammation Index; TE, tooth extraction; LA-NPC, locally advanced nasopharyngeal carcinoma; OR, odds ratio; CI, confidence interval; SE, standard error; V50, mandibular volume receiving ≥50 Gy; V60, mandibular volume receiving ≥60 Gy; df, degrees of freedom.

## Data Availability

The datasets generated and/or analyzed during the current study are available from the corresponding author on reasonable request.

## Supplementary Materials

The following supporting information can be downloaded at: https://www.mdpi.com/article/10.3390/jcm15103777/s1, Table S1: STROBE Checklist for Observational Studies. Table S2: Interaction between CRII and mandibular radiation dose parameters in relation to post-CCRT TE.

## References

[1] Blanchard P., Lee A.W.M., Marguet S., Leclercq J., Ng W.T., Ma J., Chan A.T.C., Huang P.Y., Benhamou E., Zhu G. (2015). Chemotherapy and radiotherapy in nasopharyngeal carcinoma: An update of the MAC-NPC meta-analysis. Lancet Oncol..

[2] Ribassin-Majed L., Marguet S., Lee A.W.M., Ng W.T., Ma J., Chan A.T.C., Huang P.Y., Zhu G., Chua D.T.T., Chen Y. (2017). What is the best treatment of locally advanced nasopharyngeal carcinoma? An individual patient data network meta-analysis. J. Clin. Oncol..

[3] Zhang Y., Chen L., Hu G.Q., Zhang N., Zhu X.D., Yang K.Y., Jin F., Shi M., Chen Y.P., Hu W.H. (2019). Gemcitabine and cisplatin induction chemotherapy in nasopharyngeal carcinoma. N. Engl. J. Med..

[4] Tang L.L., Guo R., Zhang N., Li W.F., Chen F.P., Sun Y., Mao Y.P., Liu L.Z., Tian L., Lin A.H. (2022). Effect of radiotherapy alone vs radiotherapy with concurrent chemoradiotherapy on survival without disease relapse in patients with low-risk nasopharyngeal carcinoma. JAMA.

[5] Walker M.P., Wichman B., Cheng A.L., Coster J., Williams K.B. (2011). Impact of radiotherapy dose on dentition breakdown in head and neck cancer patients. Pract. Radiat. Oncol..

[6] Soutome S., Yanamoto S., Funahara M., Hasegawa T., Komori T., Yamada S., Kurita H., Kojima Y., Umeda M. (2017). Risk factors for radiation-induced dental caries in patients with head and neck cancer. Oral Health Care.

[7] Gerlach T., Brunello G., Tenbrink C., Schumacher J., Haussmann J., Irschfeld L., Kämmerer P.W., Al-Nawas B., Kniha K., Holzinger D. (2026). Radiation-induced oral side effects in head and neck cancer: A scoping review and interdisciplinary recommendations. BMC Oral Health.

[8] Bhandari S., Soni B.W., Bahl A., Ghoshal S. (2020). Radiotherapy-induced oral morbidities in head and neck cancer patients. Spec. Care Dentist..

[9] Chronopoulos A., Zarra T., Ehrenfeld M., Otto S. (2018). Osteoradionecrosis of the jaws: Definition, epidemiology, staging and clinical and radiological findings. Int. Dent. J..

[10] Havndrup-Pedersen C., Møller Andersen S.W., Nyberg J., Kofod T. (2025). New classification system for osteoradionecrosis of the jaws—An integrative review. Br. J. Oral Maxillofac. Surg..

[11] Lyons A., Osher J., Warner E., Kumar R., Brennan P.A. (2014). Osteoradionecrosis—A review of current concepts in defining the extent of the disease and a new classification proposal. Br. J. Oral Maxillofac. Surg..

[12] Delanian S., Lefaix J.L. (2004). The radiation-induced fibroatrophic process: Therapeutic perspective via the antioxidant pathway. Radiother. Oncol..

[13] Marx R.E. (1983). Osteoradionecrosis: A new concept of its pathophysiology. J. Oral Maxillofac. Surg..

[14] Somay E., Topkan E., Yilmaz B., Durankus N.K., Senyurek S., Selek U. (2023). Predicting teeth extraction after concurrent chemoradiotherapy in locally advanced nasopharyngeal cancer patients using the novel GLUCAR index. Diagnostics.

[15] Yilmaz B., Somay E., Selek U., Topkan E. (2021). Pretreatment systemic immune-inflammation index predicts needs for teeth extractions for locally advanced head and neck cancer patients undergoing concurrent chemoradiotherapy. Ther. Clin. Risk Manag..

[16] Somay E., Topkan E., Bascil S., Durankus N.K., Senyurek S., Selek U. (2024). Topkan’s CARWL index efficiently predicts the radiation-induced tooth loss rates in radically treated locally advanced nasopharyngeal cancer patients. Technol. Cancer Res. Treat..

[17] Peduzzi P., Concato J., Kemper E., Holford T.R., Feinstein A.R. (1996). A simulation study of the number of events per variable in logistic regression analysis. J. Clin. Epidemiol..

[18] Riley R.D., Snell K.I.E., Ensor J., Burke D.L., Harrell F.E., Moons K.G.M., Collins G.S. (2019). Minimum sample size for developing a multivariable prediction model. Stat. Med..

[19] Schuster N.A., Rijnhart J.J.M., Twisk J.W.R., Heymans M.W., Eekhout I. (2022). Modeling non-linear relationships in epidemiology: The application and interpretation of spline models. Front. Epidemiol..

[20] Topkan E., Ekici N.Y., Ozdemir Y., Besen A.A., Yildirim B.A., Mertsoylu H., Selek U. (2019). Baseline hemoglobin <11.0 g/dL has stronger prognostic value than anemia status in nasopharynx cancers treated with chemoradiotherapy. Int. J. Biol. Markers.

[21] Tsai C.-J., Hofstede T.M., Sturgis E.M., Garden A.S., Lindberg M.E., Wei Q., Tucker S.L., Dong L. (2013). Osteoradionecrosis and radiation dose to the mandible in patients with oropharyngeal cancer. Int. J. Radiat. Oncol. Biol. Phys..

[22] von Elm E., Altman D.G., Egger M., Pocock S.J., Gøtzsche P.C., Vandenbroucke J.P. (2007). The Strengthening the Reporting of Observational Studies in Epidemiology (STROBE) statement: Guidelines for reporting observational studies. PLoS Med..

[23] Hurria A., Togawa K., Mohile S.G., Owusu C., Klepin H.D., Gross C.P., Lichtman S.M., Gajra A., Bhatia S., Katheria V. (2011). Predicting chemotherapy toxicity in older adults with cancer: A prospective multicenter study. J. Clin. Oncol..

[24] Mantovani A., Allavena P., Sica A., Balkwill F. (2008). Cancer-related inflammation. Nature.

[25] Gupta D., Lis C.G. (2010). Pretreatment serum albumin as a predictor of cancer survival: A systematic review. Nutr. J..

[26] Vaupel P., Mayer A. (2007). Hypoxia in cancer: Significance and impact on clinical outcome. Cancer Metastasis Rev..

[27] Templeton A.J., McNamara M.G., Šeruga B., Vera-Badillo F.E., Aneja P., Ocaña A., Leibowitz-Amit R., Sonpavde G., Knox J.J., Tran B. (2014). Prognostic role of neutrophil-to-lymphocyte ratio in solid tumors: A systematic review and meta-analysis. J. Natl. Cancer Inst..

[28] Hu B., Yang X.-R., Xu Y., Sun Y.-F., Sun C., Guo W., Zhang X., Wang W.-M., Qiu S.-J., Zhou J. (2014). Systemic immune-inflammation index predicts prognosis of hepatocellular carcinoma after curative resection. Clin. Cancer Res..

[29] Kratzsch T., Piffko A., Broggini T., Czabanka M., Vajkoczy P. (2020). Role of mTOR and VEGFR Inhibition in Preven-tion of Metastatic Tumor Growth in the Spine. Front. Oncol..

[30] Faulkner L., Owens J., Wadey J., Etheridge D., Lewis R. (2025). Evaluating dental dose to guide pre-radiotherapy dental extractions for various head and neck cancer sites and stages: A retrospective study. J. Radiother. Pract..

[31] Morais-Faria K., Ribeiro-Rotta R.F., Mendonça E.F., Batista A.C., Castro W.H., Mendonça A.R. (2015). Dosimetric distribution to the teeth of patients with head and neck cancer who underwent radiotherapy. Oral Surg. Oral Med. Oral Pathol. Oral Radiol..

