# Supplementary material for: The Comprehensive Repair–Inflammation Index (CRII) Predicts Tooth Extraction After Chemoradiotherapy: A Continuous and Nonlinear Modeling Analysis
Source: J Clin Med. 2026 May 14;15(10):3777. doi: 10.3390/jcm15103777 (PMC13208061; doi:10.3390/jcm15103777)
Supplement: Supplementary file 1 [file jcm-15-03777-s001.zip › jcm-4289008-supplementary.pdf]

Supplementary Table S1. STROBE Checklist for Observational Studies

Section / Item	STROBE Recommendation	Reported on Page No.
Title and Abstract		
1	Indicate the study design in the title or abstract	Title; Abstract
2	Provide an informative and balanced summary of the study	Abstract
Introduction		
3	Explain the scientific background and rationale	Introduction
4	State specific objectives and hypotheses	Introduction (final paragraph)
Methods		
5	Present key elements of study design early in the paper	Methods (Study Population)
6	Describe the setting, locations, and relevant dates	Methods (Study Population)
7	Describe eligibility criteria and selection of participants	Methods (Study Population)
8	Define outcomes, exposures, predictors, potential confounders, and effect modifiers	Methods (Endpoints and Statistical Analysis; CRII definition)
9	Describe any efforts to address potential sources of bias	Methods (multivariable adjustment, interaction analysis); Discussion (limitations)
10	Explain how the study size was arrived at	Retrospective cohort based on eligible patients; no formal a priori calculation (Methods: Statistical Analysis)
11	Explain how quantitative variables were handled in the analyses	Methods (CRII modeled as continuous; nonlinearity assessed using restricted cubic splines; exploratory categorization described separately)
12a	Describe all statistical methods, including those used to control for confounding	Methods (logistic regression, multivariable modeling, spline analysis)
12b	Describe any methods used to examine subgroups and interactions	Methods (interaction terms between CRII and mandibular dose parameters)
12c	Explain how missing data were addressed	Methods (no missing data; complete-case analysis)
12d	Describe analytical methods taking account of sampling strategy	Not applicable
12e	Describe any sensitivity analyses	Methods (nonlinearity assessed via restricted cubic splines; segmented regression used for exploratory breakpoint identification)
Results		
13a	Report numbers of individuals at each stage of study	Results (cohort description)
13b	Give reasons for non-participation at each stage	Methods (exclusion criteria)
13c	Consider use of a flow diagram	Not included
14a	Give characteristics of study participants	Results (Table 1)
14b	Indicate number of participants with missing data for each variable	Methods (no missing data; complete-case analysis)
15	Report numbers of outcome events or summary measures	Results (tooth extraction incidence)
16a	Give unadjusted and adjusted estimates with precision (e.g., 95% CI)	Results (Table 2)
16b	Report category boundaries when continuous variables were categorized	Results (CRII ≥145.7 exploratory cutoff)
16c	If relevant, translate estimates into clinically meaningful measures	Results (per 10-unit OR; predicted probabilities)
17	Report other analyses (e.g., subgroup, interaction, nonlinear analyses)	Results (spline analysis; interaction analysis)
Discussion		

18	Summarize key results with reference to study objectives	Discussion (opening paragraph)
19	Discuss limitations, including potential sources of bias	Discussion (limitations section)
20	Give a cautious overall interpretation of results	Discussion
21	Discuss the generalizability of the results	Discussion
Other Information		
22	Give the source of funding and the role of funders	Declarations / Funding statement

Supplementary Table S2. Interaction between CRII and mandibular radiation dose parameters in relation to post-CCRT TE.

Interaction term	OR	95% CI	p-value
CRII × Dmean	1.0007	0.9996–1.0017	0.233
CRII × V50	0.991	0.963–1.019	0.520
CRII × V60	0.992	0.950–1.035	0.700

Note: Interaction terms between CRII and mandibular radiation dose parameters were evaluated using logistic regression. No significant interaction was observed for mandibular mean dose, V50 (≥1 cc), or V60 (≥1 cc), indicating no evidence of effect modification by radiation dose. Results are presented as odds ratios (ORs) with 95% confidence intervals (CIs).
